# The Synergistic Effect of Iodide and Sodium Nitrite on the Corrosion Inhibition of Mild Steel in Bicarbonate–Chloride Solution

**DOI:** 10.3390/ma9110868

**Published:** 2016-10-26

**Authors:** Gaius Debi Eyu, Geoffrey Will, Willem Dekkers, Jennifer MacLeod

**Affiliations:** Department of Chemistry, Physics and Mechanical Engineering, Queensland University of Technology, Brisbane QLD 4000, Australia; g.will@qut.edu.au (G.W.); w.dekkers@qut.edu.au (W.D.); jennifer.macleod@qut.edu.au (J.M.)

**Keywords:** mild steel, bicarbonate solution, polarization, EIS, iodide ion, passivation

## Abstract

The effect of potassium iodide (KI) and sodium nitrite (NaNO_2_ inhibitor on the corrosion inhibition of mild steel in chloride bicarbonate solution has been studied using electrochemical techniques. Potentiodynamic polarisation data suggest that, when used in combination, KI and NaNO_2_ function together to inhibit reactions at both the anode and the cathode, but predominantly anodic. KI/NO_2_^−^ concentration ratios varied from 2:1 to 2:5; inhibition efficiency was optimized for a ratio of 1:1. The surface morphology and corrosion products were analysed using scanning electron microscopy (SEM) and X-ray diffractometry (XRD). The latter shows that the addition of I^−^ to NO_2_ facilitates the formation of a passivating oxide (γ-Fe_2_O_3_) as compared to NO_2_^−^ alone, decreasing the rate of metal dissolution observed in electrochemical testing. The synergistic effect of KI/NO_2_^−^ inhibition was enhanced under the dynamic conditions associated with testing in a rotating disc electrode.

## 1. Introduction

Bicarbonate-induced corrosion is potentially relevant in many applications, such as wellbore systems for geological sequestration of CO_2_ [1], and coal seam gas infrastructure. Studies indicate that chloride [2] and HCO_3_^−^ concentrations are correlated with carbon steel dissolution [3,4,5], with consequences for service life. Many methods such as cathodic protection, phosphating, coating and the use of inhibitors [6,7,8,9] have been adopted to reduce corrosion. Among them, the use of an inhibitor is one of the most popular, economic, convenient and practical measures for corrosion control in certain situations [10]. Inorganic inhibitors such as nitrite, phosphate and chromate have been used in acidic solutions and industrial water. However, the use of chromates has been restricted in recent years because of their toxic nature. Sodium nitrite has been used as corrosion inhibitor in gasoline and petroleum product pipelines [11], but it can be toxic in large amounts [12]. Nitrites retard anodic reactions by oxidizing ferrous ions to ferric ions at the steel surfaces to produce a stable tenacious film of iron (III) oxide [13] as:

Fe → Fe^2+^ + 2e^−^ (1)

Fe^2+^ + 2OH^−^ + 2NO_2_^−^ → 2NO + γ-Fe_2_O_3_ (2)

The resulting complex (2NO + γ-Fe_2_O_3_) can either inhibit or catalyse corrosion, depending on its relative stability. Nitrite raises the critical breakdown potential and the potential at the steel surface, and when the concentration is sufficient, the steel is moved into the passive region corresponding to the Fe_2_O_3_ region in the Pourbaix diagram. Conversely, the corrosion rate can be increased when the nitrite concentration is below a critical level [14]. Raman and Siew also reported that NO_2_^−^ has a concentration-dependent effect on the susceptibility of stainless steel to stress corrosion cracking (SCC) when chloride is present. They find that SCC was significantly decreased at low nitrite concentrations (1400 and 2800 ppm), but that it increases considerably at nitrite concentration of 5600 ppm [15].

In recent years, the performance of inhibitors has been enhanced by the addition of either organic or inorganic compounds [16]. For example, Oguzie et al. [17] studied the synergistic corrosion inhibition of methionine and potassium iodide in acidic media. They concluded that potassium iodide enhanced methionine inhibition significantly when present in a 5:5 ratio. Pavithra et al. [18] also reported that the addition of potassium iodide to benzisothiozole-3-piperizine hydrochloride improved the corrosion resistance of mild steel in acid conditions. To date, the research focus has been on the synergistic effect of potassium iodide and organic inhibitors. There are no studies of the influence of KI on inorganic inhibitors on the corrosion of steel in both acidic and alkaline media under stagnant and dynamic conditions. The present study investigates the effect of potassium iodide and its synergistic effect on sodium nitrite inhibition on the corrosion of mild steel in high bicarbonate-chloride solution under stagnant and dynamic conditions. It is anticipated that the outcome of this study will enhance the applications of sodium nitrite. 

## 2. Results and Discussion

### 2.1. Effect of Sodium Nitrite Concentration

**Figure 1:**
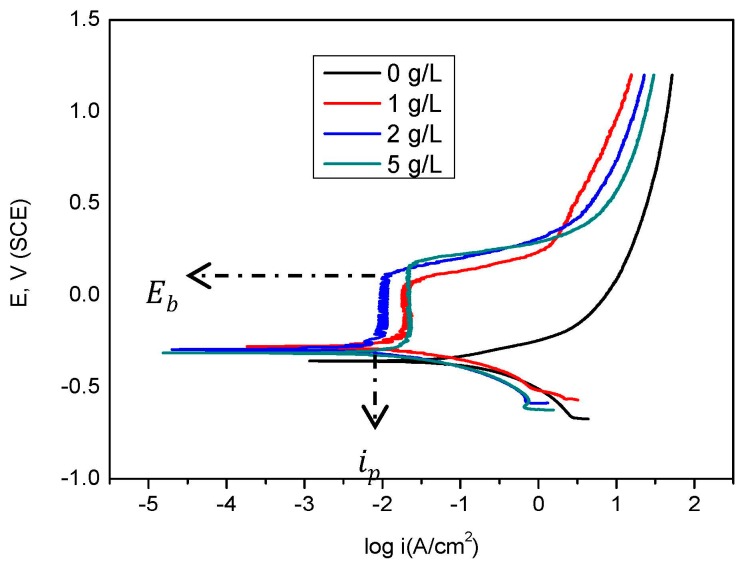
Polarization curves for mild steel in bicarbonate/chloride solution in absence and presence of different concentration of sodium nitrite at 2000 rpm at room temperature.

Figure 1 shows the polarisation curve for mild steel in bicarbonate/chloride solution containing different nitrite concentrations. The anodic regime was apparently active in solutions without nitrite, while passive behaviour occurred in solutions containing nitrite. Passivation is eliminated in the absence of sodium nitrite due to chloride ions at the metal surface, which is responsible for the anodic sensitivity [19,20] and thus, an increase in anodic current density. 

The breakdown potential increases with nitrite concentrations. The anodic current densities decreased with an increase in nitrite concentration, resulting in a decrease in corrosion current densities. The results are consistent with the formation of a passive film, which slows down the corrosion rate [14,21]. We found that, over most of the tested range, the passive current density ($i_{p}$) decreases with increasing nitrite concentrations. However, the achieved inhibition efficiency reached an optimum value at 2 g/L, and a further nitrite increase in nitrite concentration to 5 g/L resulted in a decrease in efficiency. Together, these results suggest that, when nitrite concentration is below the critical level, the steel surface is partially passivated and when the concentration is above the optimum level, the ionic conductivity of the solution is raised, increasing the corrosion rate [21]. Generally, nitrite repairs the oxide layer on the steel/solution interface. The inhibition mechanism is based on the oxidation of iron (II) to iron (III) oxide. Oxide film nucleation is initiated when the Fe (II) cations combine with H_2_O molecules. The process involves reduction and oxidation of nitrite and Fe^2+^, respectively, as:

2NO_2_^−^ + 4e^−^ → N_2_O + 3O^2−^ (reduction) (3)

Fe → Fe^2+^ + 2e^−^ (oxidation) (4)

Fe^2+^ + 2OH^−^ + 2NO_2_^−^ → 2NO + γ-Fe_2_O_3_ (5)

The reduction of the nitrite ions at the steel surface promotes the release of O^2−^. These O^2−^ ions oxidize the ferrous ion produced on the steel surface to form an insoluble and a stable barrier layer (γ-Fe_2_O_3_) [22] over the steel surface. 

**Figure 2:**
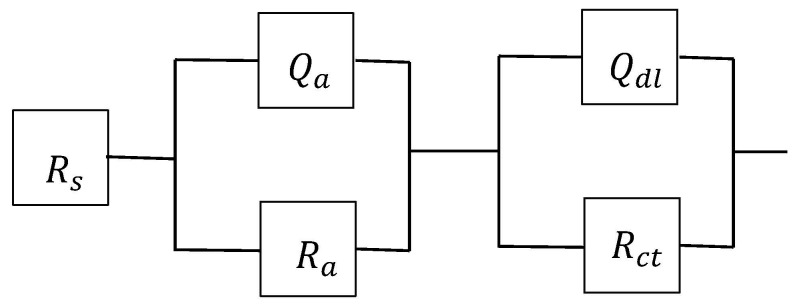
Equivalent circuit for impedance measurements.

**Figure 3:**
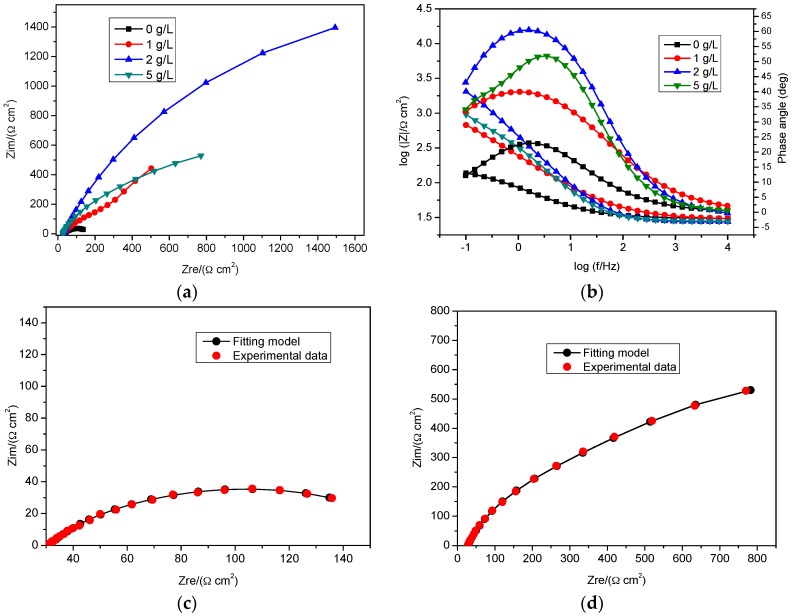
Impedance spectra for mild steel in bicarbonate chloride solution in the absence and presence of sodium nitrite at 2000 rpm at room temperature: (**a**) Nyquist plots and (**b**) Bode and impedance magnitude vs. frequency plots; (**c**) Nyquist plots (0 g/L nitrite) and (**d**) Nyquist plots (5 g/L nitrite) for fitted and experimental data.

Impedance spectra were obtained from a rotating disk electrode (specimen) in bicarbonate/chloride solutions with and without inhibitors. The spectra were fitted with an equivalent electrical circuit model as shown in Figure 2, and it showed good agreement with the experimental data (Figure 3c,d), where R_s_, R_ct_, R_a_, Q_dl_ and Q_a_ represent the solution resistance, charge transfer resistance (metal/film interface), adsorption resistance (solution/film interface), double layer capacitance and passive film capacitance, respectively. The use of constant phase element (CPE) defined by the values of n and Q, is commonly used to compensate for inhomogeneity of electrode surface. The impedance, Z of the constant phase element as given in [23]:

Z_CPE_ = Q^−1^(jω)^−n^ (6)

where Q and n are constant and exponent, respectively, j = ($\sqrt{-1}$) is an imaginary number, ω=2πf is the angular frequency in rad/s calculated using f, the frequency in H_z_. At low frequencies, the impedance and phase angles describes the kinetic response for the charge transfer activity, while at high frequencies they depend on surface layer inhomogeneity [24].

Figure 3a,b represent the Nyquist and a combined Bode-phase angle plots in the presence and absence of nitrite at 2000 rpm. The addition of an inhibitor results in an increase in impedance, charge transfer resistance and a reduction of double layer capacitance as shown in Table 1. This implies that, the adsorption of the inhibitor at the metal/electrolyte interface enhances the formation of a stable oxide film and, consequently, results in a decrease in corrosion rate. The phase angle maxima increase with increasing nitrite concentration is either due to homogeneity of the absorbed inhibitor on the metal surface or to a reduction in porosity of the passive film [25]. The inhibitor efficiency was calculated according to Equation (18), and revealed the highest inhibition efficiency of 96.40% at 2 g/L. A further increase to 5 g/L reduces inhibitor efficiency to 89.22% in agreement with the data obtained from the polarization curves.

### 2.2. Effect of Potassium Iodide Additives

The corrosion of steel in aqueous solution is known to be significantly exacerbated by hydroxyl ions, which form intermediate catalytic complexes on the steel surface [26]:

Fe + OH^−^ ⇌ (FeOH)_ads_ + e (7)

(FeOH)_ads_ → FeOH^−^ + e (8)

FeOH^+^ + H^+^ ⇌ Fe^2+^ + H_2_O (4c) (9)

The replacement of the absorbed hydroxyl ions on the metal surface by halide ions can decrease the hydroxyl catalytic effect [27]:

Fe + H_2_O + Y^−^ ⇌ (FeYOH)^−^_ads_ + H^+^ + *e* (10)

(FeYOH)^−^_ads_ → FeYOH + *e* (11)

FeYOH + H^+^ ⇌ Fe^2+^ + Y^−^ + H_2_O (12)

where Y represents a halide ion. This replacement ability follows the trend $I^{-}$ > ${\mathrm{Br}}^{-}$ > ${\mathrm{Cl}}^{-}$. The greater effect of the iodide ion is attributed to its high hydrophobicity, low electronegativity and relatively large ionic radius [17], which enables $I^{-}$ ions to form a stronger bond with the iron surface. 

**Figure 4:**
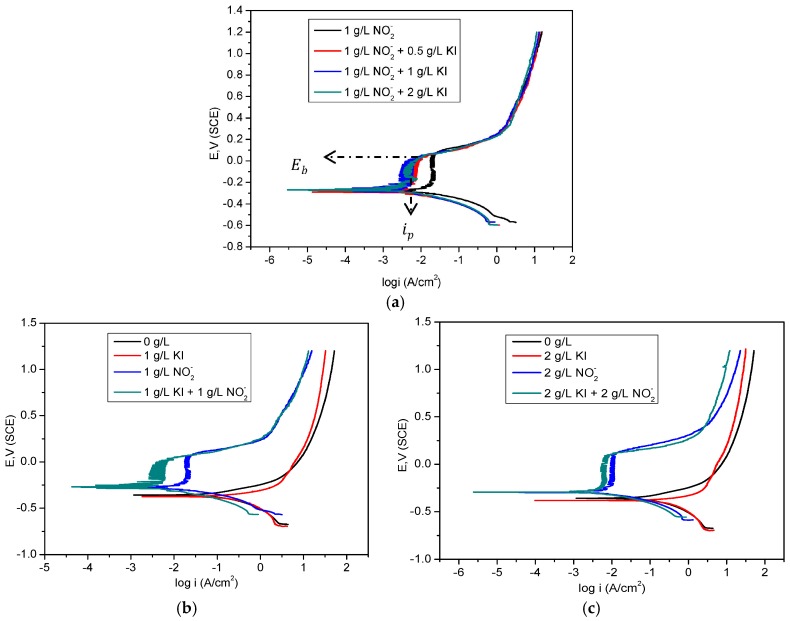
Polarization curves for mild steel in bicarbonate/chloride solution at 2000 rpm at room temperature: (**a**) in absence and presence of different concentrations of KI (**b**,**c**).

Figure 4a–c show the polarisation curves for corrosion of mild steel in the bicarbonate/chloride solutions with and without KI at the indicated concentration. The data shows that KI addition reduce both the rates of anodic Fe dissolution and cathodic hydrogen evolution. As reported by Heusler and Cartledge [28], the $I^{-}$ ion inhibits both the anodic and cathodic reactions, but predominantly affects the anodic site. The increase in corrosion potential (*E*_corr_) and decrease in corrosion rate with increasing KI concentration are similar to those reported previously [17,29]. The lowest passive current densities $\left(i_{p}\right)$ were obtained at KI/NO_2_^−^ = 1/1 and 2/2 as depicted in Figure 4b,c. Excessive quantities of KI increase the passivation current due to limited active site available for inhibitor adsorption [30]. Besides the replacement of the hydroxyl ions on the metal surface by halide ions, the chemisorption of I^−^ ions can increase the hydrophobicity of the metal surface [31]:

FeIOH + H^+^ ⇌ Fe^2+^ + I^−^ + H_2_O (13)

Fe^2+^ + I^−^ + 2OH^−^ + 2NO_2_^−^ → 2NO + γ-Fe_2_O_3_ + I^−^ (14)

The iodide reacts with oxygen to form molecular iodine (I_2_) [32] which reacts with the oxidized steel to form FeI_2(solid)_ [33] which could increase the stability of the oxide film. The adsorption of iodide molecules on the steel surface involves chemisorption [34,35]. The iodide ion slows down cathodic reaction, raising the hydrogen overpotential, whereas at the anode, its influence may act as a barrier to the hydroxyl ion, whose presence at the metal/solution interface is necessary for corrosion process [36].

**Figure 5:**
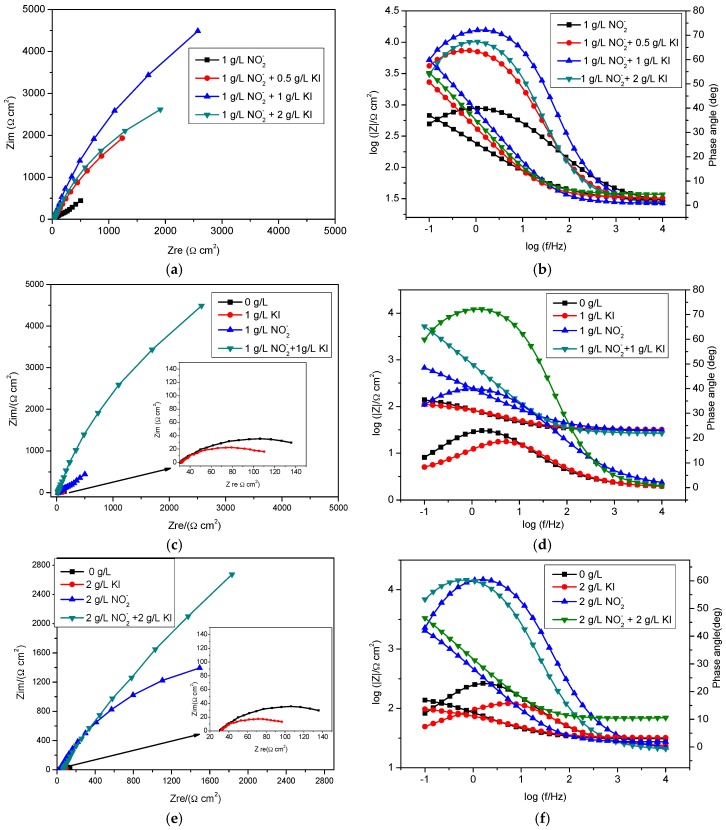
Impedance spectra for mild steel in bicarbonate/chloride solution at 2000 rpm at room temperature: (**a**,**b**) Nyquist, Bode and impedance magnitude plots in absence and presence of (**c**,**d**) different concentrations of KI and (**e**,**f**) absence and presence of KI.

The Nyquist and Bode Phase angle plots shown in Figure 5a–f, for bicarbonate/chloride solution in the absence and presence of different concentrations of KI additives, reveal that the addition of KI leads to a significant increase in the diameters of the semi-circles in the Nyquist plots and in the maxima phase angle in the Bode plots. The diameters and phase angle maxima increase with increasing concentrations of KI from 0.5 to 1 g/L, indicating that increasing KI concentration promotes inhibitor action. However, a further increase of KI concentration to 2 g/L results in a decrease in both the diameter of the semi-circles in Nyquist plots and phase angle maxima, as shown in Figure 5a,b. These same trends are apparent in the charge transfer resistance given in Table 2 and Table 3. The results suggest that more inhibitive passive films were achieved when the concentrations of both nitrites and KI are similar. 

### 2.3. Effect of Flow Velocity

**Figure 6:**
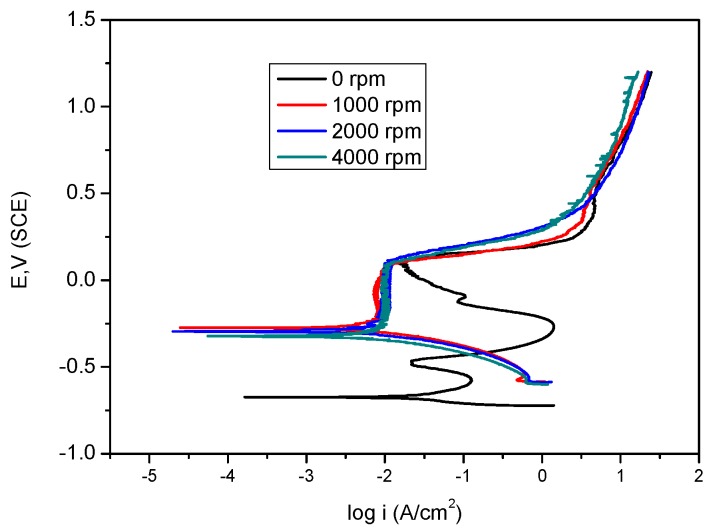
Polarization curves for mild steel in bicarbonate/chloride solution under stagnant conditions and different rotation speeds in the presence of 2 g/L NO_2_^−^ + 2 g/L KI at room temperature.

The synergistic effect of KI was studied under stagnant and various rotation speed conditions. The polarisation profile shows that passivation was achieved under flow conditions, whereas under stagnant conditions, two anodic peaks were apparent as illustrated in the polarisation profile shown in Figure 6. This implies that high electrode rotation speed facilitates film formation on the metal surface [37,38]. On the other hand, an unstable non-protective oxide film was formed under stagnant conditions, and as a result metal dissolution was uninhibited. It is apparent that the anodic current densities decrease while corrosion potentials become more noble [39] with increasing rotation speeds. $I^{-}$ promotes the replacement of hydroxyl ions, facilitating the adsorption of inhibitor to the metal surface:

Inh_(sol)_ + yH_2_O_(ads)_ ⇌ Inh_(ads)_ + yH_2_O_(sol)_ (15)

The inhibitor combines with freshly formed Fe^+^ ions on the steel surface, forming inhibitor metal complexes [40]:

F → Fe^2+^ + 2e (16)

Fe^2+^ + Inh_(ads)_ → (Fe-Inh)^2+^_(ads)_ (17)

The resulting complexes can either inhibit or catalyse further metal dissolution, depending on their relative stability. Flow has both positive and negative influences on inhibitor efficiency; it increases mass transport of inhibitor molecules and oxygen to the steel surface, which can improve the inhibition performance. On the other hand, high velocity flow can increase mass transport of Fe^2+^ ions formed during metal dissolution away from the electrode surface to the bulk of solution. This impedes the formation of metal/inhibitor complexes on the electrode and further increase metal dissolution [41]. In our study, more (Fe-Inh)^2+^ complex was formed on the electrode under dynamic conditions as compared to stagnant conditions. The increased passivation was apparent in the polarization curves.

**Figure 7:**
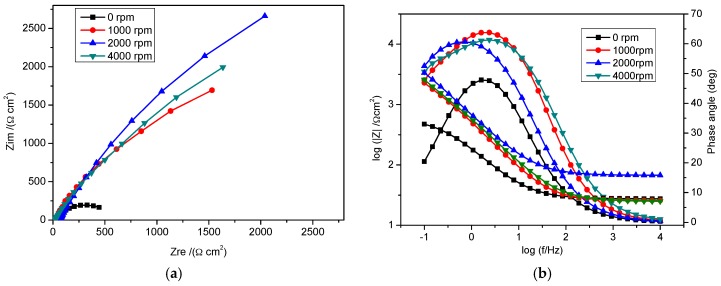
Impedance spectra for mild steel in bicarbonate chloride solution under stagnant and indicated rotating speeds in the presence of 2 g/L NO_2_^−^ + 2 g/L KI at room temperature: (**a**) Nyquist plots and (**b**) Bode and impedance magnitude vs. frequency plots.

Impedance spectra are shown in Figure 7. The Nyquist plots (Figure 7a) show that the semi-circle increases with increasing rotation speed (0–2000 rpm). This result implies that a more stable film forms as rotation speed increases. The passive films on the surface causes an increase in charge transfer resistance and a decrease in double layer capacitance [12]. However, at a higher rotation speed (4000 rpm), the charge transfer resistance decreases due to depletion of the (Fe-Inh)^2+^ complex, and the corrosion rate increases. The steel surface resistance increases with rotation speed as revealed in the Bode and impedance magnitude in Figure 7b at low frequencies. The summaries of the EIS parameters are listed in Table 4. The result is in agreement with the polarization data obtained.

### 2.4. SEM and EDS Analysis

**Figure 8:**
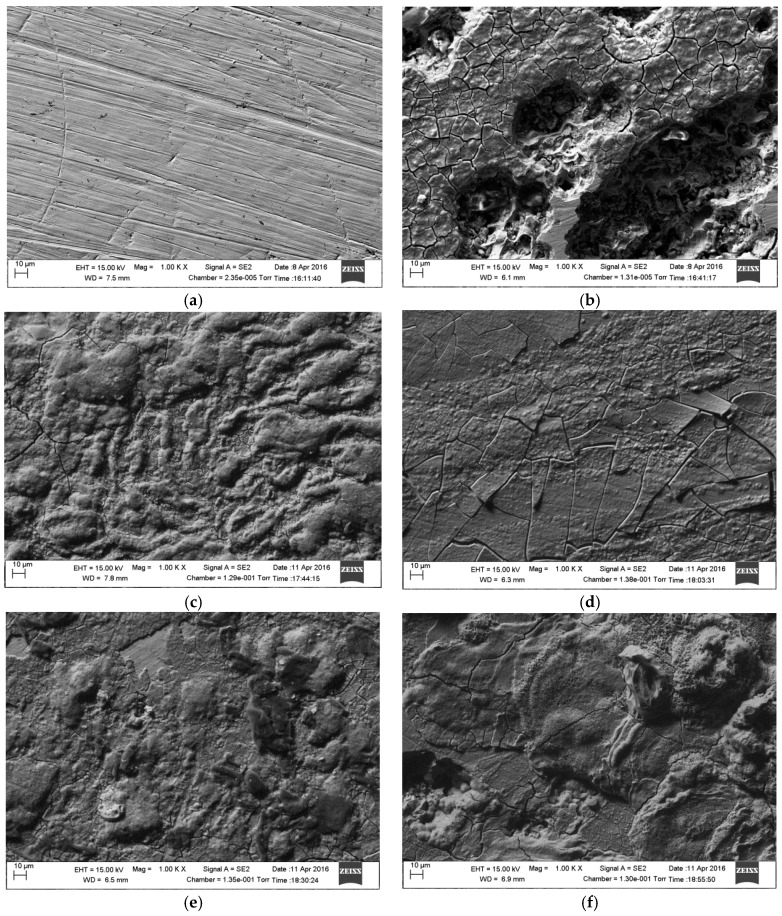
Scanning electron micrographs of mild steel surface before and after corrosion testing: (**a**) before immersion; (**b**) without inhibitor; (**c**) 1 g/L NO_2_^−^; (**d**) 1 g/L NO_2_^−^ + 0.5 g/L KI; (**e**) 1 g/L NO_2_^−^ + 1 g/L KI; (**f**) 1 g/L NO_2_^−^ + 2 g/L KI.

**Figure 9:**
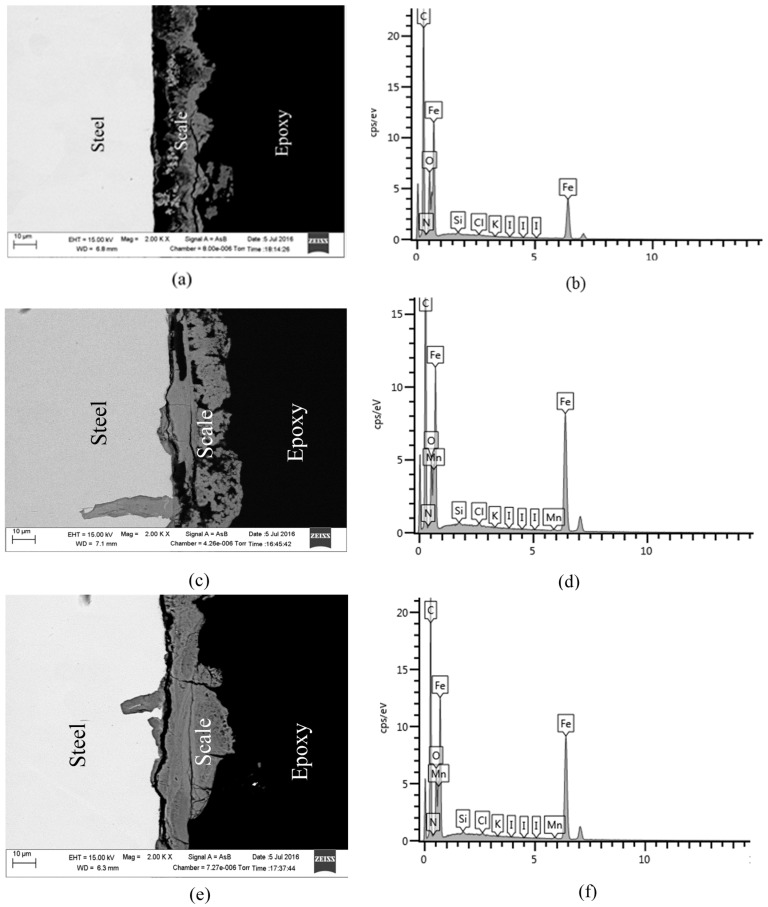
SEM cross-sectional images and EDS spectra after corrosion tests: (**a**,**b**) un-inhibited (**c**,**d**) nitrites (**e**,**f**) nitrites + KI containing solutions.

The surface morphology of the test specimens in the presence and absence of KI additives was investigated using scanning electron microscopy as shown in Figure 8 and the elemental composition of the corrosion product was analysed using EDS. Figure 8a shows the surface morphology before corrosion testing. It is obvious that specimens immersed in solution without nitrite or KI additives shows significant surface deterioration, and pitting corrosion is evident in the surface morphology (Figure 8b). The addition of 1 g/L of nitrite to the solution decreases the corrosion, but a few cracks have formed on the corrosion product on the steel surface (Figure 8c). In presence of combined NO_2_^−^ with KI (0.5, 1, and 2 g/L), the steel specimens exhibit less corrosion damage and retain, relatively smooth surfaces (Figure 8c–e). This indicates that the adsorption of $I^{-}$ ions on mild steel expedites the formation of more stable films. The oxide film is presumably more adhered to the steel surface in the presence of $I^{-}$ ions, and as a result impedes iron dissolution. However, a few cracks are observed when KI concentration was increased to 2 g/L. Figure 9 shows the cross-sectional SEM images of corrosion products (Figure 9a,c,e) and their corresponding EDS spectra (Figure 9b,d,f) taken after the corrosion tests. The presence of inhibitor influences the corrosion product morphologies. The addition of KI improves the compactness and the stability of the corrosion product on the steel surface as shown in Figure 9c. This implies that the diffusion of Fe^2+^ from the steel and the ingress of corrosive ions to the steel surface were inhibited. 

### 2.5. XRD Analysis

**Figure 10:**
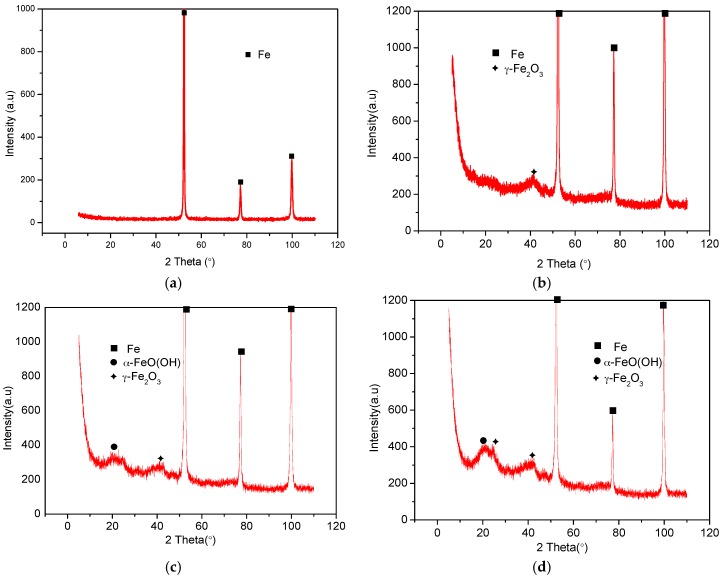
XRD analysis of corrosion product after corrosion test of mild steel in bicarbonate/chloride solution at 2000 rpm: (**a**) un-immersed (**b**) un-inhibited (**c**) 1 g/L NO_2_^−^ (**d**) 1 g/L NO_2_^−^ + 1 g/L KI.

XRD patterns in Figure 10b–d, show the different diffraction peaks associated with surfaces tested in solutions without inhibitor, with 1 g NO_2_^−^ and 1 g/L NO_2_^−^ + 1 g/L Kl, respectively. The figures show similar peak patterns, α-FeO (OH) (goethite) and γ-Fe_2_O_3_ (maghemite) were the iron oxyhydroxides present in the corrosion products. Goethite peak was not present in the uninhibited specimen as shown in Figure 10b. The formation of goethite is mainly attributed to the oxidation of γ-Fe_3_O_4_ by atmospheric O_2_ after removal from the test solutions. Figure 10a shows the pattern of the specimen before corrosion test.

## 3. Experimental

### 3.1. Materials and Solutions

Cylindrical carbon steel rod (Ø16 mm) of grade AISI 1020 was used as the rotating disc electrode (RDE). The chemical composition of the as-received rod was analysed using a Field Emission Electron Probe Microanalyzer (JXA-8530 F) (JEOL, Peabody, MA, USA) as shown in Table 5. The test samples were polished using emery papers of grit sizes ranging from 220 to 800, rinsed with acetone and dried with air. The sample preparation procedures for all specimens were completed within 8 min prior to the subsequent electrochemical tests, to ensure an identical surface oxide film for all specimens. The solution was prepared by dissolving analytical grade sodium bicarbonate (5 g) and 2 g of sodium chloride in 1 L of distilled deionized water which corresponds to 0.06 M and 0.03 M bicarbonate and chloride concentrations, respectively. All experiments were conducted in solution containing different concentrations of nitrite (0–5 g/L) and potassium iodide (0–2 g/L) to study the influence of sodium nitrite and potassium iodide, respectively. The electrochemical tests were performed at ambient temperature (22 ± 3 °C) at pH (8.2 ± 0.1).

### 3.2. Electrochemical Test

The corrosion tests were carried out in a 1 L glass cell containing the test solution (400 mL) and a rotating disk electrode (working electrode) fitted into rotating shaft of an analytical rotator AFASRE 747 (Pine Instrument Company, Grove, PA, USA). The unexposed surface area was embedded in epoxy, leaving exposed area of 2.01 cm^2^ in contact with the corrosive solution. The reference electrode was a saturated calomel electrode (SCE) connected to the cell by a Lugging capillary, and platinum was used as counter electrode. The angular speed (ω) of the rotating disk electrode was controlled by the ASR RDE speed controller. The minimum and maximum rotation speed of the RDE performed in the tests was 1000 and 4000 rev/min, respectively. Corrosion evaluation was monitored by potentiodynamic polarization and electrochemical impedance spectroscopy. Before electrochemical tests, the working electrode was immersed in test solution at open circuit potential (OCP) for 30 min to attain a stable state. Electrochemical impedance spectroscopy (EIS) measurements were conducted at open-circuit. The measuring frequencies ranged from 10 kHz to 0.1 Hz with a perturbing alternating current (AC) amplitude of 10 mV and a sampling rate of 10 points per decade. Polarization studies were performed after EIS tests at room temperature at a scan rate of 0.5 mV/s relative to the open circuit corrosion potential using a Bio-Logic instrument Model VSP 0508 potentiostat (BioLogic, Knoxville, TN, USA). The potentiodynamic polarisation was obtained from a starting potential of −0.30 V vs. SCE to a final potential of +1.20 V vs. SCE. The results obtained from EIS were analysed and fitted to equivalent circuits using Z Fit contained in the EC-Lab^®^ [23]. Data obtained from EIS were used to calculate the inhibition efficiency (η) as [42]:

(18) $$\eta \left(\%\right)=\;\frac{R_{ct\left(inh\right)}-R_{ct}}{R_{ct\left(inh\right)}}\times \;100$$

where $R_{ct\left(inh\right)}$ and $R_{ct}$ are charge transfer resistance in the presence and absence of inhibitors, respectively. The degree of surface coverage (θ) was calculated using the relationship:

θ = η(%)/100 (10)
(19)

### 3.3. Scanning Electron Microscopy (SEM)

Scanning electron microscopy (SEM) imaging were performed before and after corrosion tests using a Zeiss Sigma VP Field Emission Scanning Electron Microscope (Oxford XMax 50 with silicon drift energy dispersive spectroscopy (EDS) detector (Jena, Germany)), in secondary electron (SE) imaging mode with an accelerating beam voltage of 15 kV. For cross-section analyses, specimens were cut and mounted with epoxy resin. The surface was polished with alumina 0.05 μm without etching and fine carbon films were sputtered on the specimens to reduce charging of the epoxy resin.

### 3.4. X-ray Diffraction

The corrosion products were characterized using a PANalytical X’ Pert PRO MPD powder X-ray Diffractometer (Almelo, The Netherlands), 40 keV, 40 mA, (Co K_α_) with the multi-purpose vertical stage and a 5 mm mask in Bragg-Brentano geometry. Data were collected from 2θ values of 5°–110° at a step size of 0.0084°.

## 4. Conclusions

This study examined the corrosion inhibition of sodium nitrite and the synergistic effect of potassium iodide on the corrosion behaviour of mild steel in high concentration bicarbonate chloride solution. Sodium nitrite functions as an anodic inhibitor, and the inhibition efficiency was significantly improved by the addition of $I^{-}$. The data suggest a mixed-inhibition mechanism, with a dominant effect on the anodic reaction, based on the formation of a passivating oxide (γ-Fe_2_O_3_) that decreases the rate of metal dissolution, is responsible for the effective inhibition. Corrosion inhibition efficiency was optimum at NO_2_^−^/KI = unity. The synergistic effect of KI was due to the displacement of hydroxyl ions and the presence of nitrite in the solution, which facilitates the oxidation of iron in the divalent state (Fe_3_O_4_) to form a stable tenacious trivalent oxide (γ-Fe_2_O_3_) on the steel/solution interface. Inhibition efficiency increased with rotation speeds (0–2000 rpm) due to rapid oxide film formation. However, a further increase of rotation speed (4000 rpm), expedites the removal of (Fe^2+^-Inh)_(ads)_ from the steel surface and accelerates metal dissolution. These results suggest that sodium nitrite inhibitor can be made more environmentally friendly and effective when synergised with potassium iodide, since smaller amounts of nitrite are required when used in conjunction with KI, which is a non-toxic compound to both human and animals.

## Figures and Tables

**Table 1:** Impedance parameters for mild steel in bicarbonate/chloride solution in the absence and presence of different concentration of sodium nitrite at 2000 rpm at room temperature.

Inh. Conc (g/L)	R_s_ (Ω·cm^2^)	R_ct_ (Ω·cm^2^)	Q_dl_ (F·cm^−2^)	*n*	R_a_ (Ω·cm^2^)	Q_a_ (F·cm^−2^)	η (%)	Surface Coverage (θ)
0	58.41	160.8	4.37 × 10^−3^	0.59	29.96	1.26 × 10^−3^	0	0
1g NO_2_^−^	18.54	2048	1.51 × 10^−3^	0.55	10.68	5.0 × 10^−1^	92.14	0.921
2g NO_2_^−^	15.33	4473	5.22 × 10^−4^	0.76	11.06	1.02 × 10^−4^	96.40	0.964
5g NO_2_^−^	27.80	1492	1.59 × 10^−3^	0.78	303.5	9.4 × 10^−4^	89.22	0.892

**Table 2:** Impedance parameters for mild steel in bicarbonate/chloride solution in the absence and presence of different concentrations of KI at 2000 rpm at room temperature.

Inh. Conc (g/L)	R_s_ (Ω·cm^2^)	R_ct_ (Ω·cm^2^)	Q_dl_ (F·cm^−2^)	n	R_a_ (Ω·cm^2^)	Q_a_ (F·cm^−2^)	η (%)	Surface Coverage (θ)
0	58.41	160.8	4.37 × 10^−3^	0.59	29.96	1.26 × 10^−3^	0	0
1KI	31.74	176.7	4.09 × 10^−2^	0.22	65.92	2.60 × 10^−3^	8.99	0.089
1NO_2_^−^	29.57	1911	1.49 × 10^−3^	0.56	8.66	1.97 × 10^−5^	91.58	0.916
1NO_2_ + 0.5KI	22.50	10,621	5.70 × 10^−4^	0.78	335.40	5.61 × 10^−3^	98.48	0.985
1NO_2_ + 1KI	26.94	12,009	3.76 × 10^−4^	0.99	2156	5.31 × 10^−4^	98.66	0.987
1NO_2_ + 2KI	93.70	9029	3.85 × 10^−4^	0.83	163.0	1.30 × 10^−3^	98.21	0.982

**Table 3:** Impedance parameters for mild steel in bicarbonate/chloride solution in the absence and presence of KI at 2000 rpm at room temperature.

Inh. Conc (g/L)	R_s_ (Ω·cm^2^)	R_ct_ (Ω·cm^2^)	Q_dl_ (F·cm^−2^)	n	R_a_ (Ω·cm^2^)	Q_a_ (F·cm^−2^)	η (%)	Surface Coverage (θ)
0	58.41	160.8	4.37 × 10^−3^	0.59	29.96	1.26 × 10^−3^	0	0
2KI	31.81	46.32	1.47 × 10^−2^	0.59	33.97	3.30 × 10^−3^	−71.19	−0.712
2NO_2_^−^	15.53	4473	5.22 × 10^−4^	0.76	11.06	1.02 × 10^−4^	96.40	0.964 oo
2NO_2_ + 2KI	22.30	13,347	3.78 × 10^−4^	0.75	78.78	0.9 × 10^−4^	98.79	0.988

**Table 4:** Impedance parameters for mild steel in bicarbonate/chloride solution under stagnant conditions and different rotation speeds in the presence of 2 g/L NO_2_^−^ + 2 g/L KI at room temperature.

Rotation Speed (rpm)	R_s_ (Ω·cm^2^)	R_ct_ (Ω·cm^2^)	Q_dl_ (F·cm^−2^)	n	R_a_ (Ω·cm^2^)	Q_a_ (F·cm^−2^)
0	27.45	508.3	1.26 × 10^−3^	0.76	61.01	1.79 × 10^−4^
1000	25.61	3597	1.14 × 10^−3^	1	1536	5.60 × 10^−4^
2000	22.3	13,347	3.78 × 10^−4^	0.75	78.79	0.92 × 10^−4^
4000	24.87	3859	3.4 × 10^−4^	1	3771	3.92 × 10^−4^

**Table 5:** Chemical composition of mild steel (wt %).

C	Si	Mn	Cr	Cu	Ni	Fe
0.20	0.32	0.79	0.01	0.01	0.01	Bal 98.7
